# Deletion of the EP402R Gene from the Genome of African Swine Fever Vaccine Strain ASFV-G-∆I177L Provides the Potential Capability of Differentiating between Infected and Vaccinated Animals

**DOI:** 10.3390/v16030376

**Published:** 2024-02-28

**Authors:** Manuel V. Borca, Elizabeth Ramirez-Medina, Nallely Espinoza, Ayushi Rai, Edward Spinard, Lauro Velazquez-Salinas, Alyssa Valladares, Ediane Silva, Leeanna Burton, Amanda Meyers, Jason Clark, Ping Wu, Cyril G. Gay, Douglas P. Gladue

**Affiliations:** 1 Foreign Animal Disease Research Unit, Plum Island Animal Disease Center, U.S. Department of Agriculture, Agricultural Research Service, Orient, NY 11957, USA; elizabeth.ramirez@usda.gov (E.R.-M.); nallely.espinoza@usda.gov (N.E.); ayushi.rai@usda.gov (A.R.); edward.spinard@usda.gov (E.S.); lauro.velazquez@usda.gov (L.V.-S.); alyssa.valladares@usda.gov (A.V.); amanda.meyers@usda.gov (A.M.); 2 Foreign Animal Disease Research Unit, U.S. Department of Agriculture, Agricultural Research Service, National Bio and Agro-Defense Facility, Manhattan, KS 66502, USA; ediane.silva@usda.gov (E.S.); leeanna.burton@usda.gov (L.B.); jason.clark@usda.gov (J.C.); 3 Oak Ridge Institute for Science and Education (ORISE), Oak Ridge, TN 37830, USA; 4 Plum Island Animal Disease Center, U.S. Department of Agriculture, Animal and Plant Health Inspection Service, Orient, NY 11957, USA; ping.wu@usda.gov; 5 Agricultural Research Service, U.S. Department of Agriculture, Beltsville, MD 20705, USA; cyril.gay@usda.gov

**Keywords:** African swine fever virus, African swine fever, ASF vaccine, EP402R marker vaccine, I177L, Ep402R

## Abstract

The African swine fever virus (ASFV) mutant ASFV-G-∆I177L is a safe and efficacious vaccine which induces protection against the challenge of its parental virus, the Georgia 2010 isolate. Although a genetic DIVA (differentiation between infected and vaccinated animals) assay has been developed for this vaccine, still there is not a serological DIVA test for differentiating between animals vaccinated with ASFV-G-∆I177L and those infected with wild-type viruses. In this report, we describe the development of the ASFV-G-∆I177L mutant having deleted the *EP402R* gene, which encodes for the viral protein responsible for mediating the hemadsorption of swine erythrocytes. The resulting virus, ASFV-G-∆I177L/∆EP402R, does not have a decreased ability to replicates in swine macrophages when compared with the parental ASFV-G-∆I177L. Domestic pigs intramuscularly (IM) inoculated with either 10^2^ or 10^6^ HAD_50_ of ASFV-G-∆I177L/∆EP402R remained clinically normal, when compared with a group of mock-vaccinated animals, indicating the absence of residual virulence. Interestingly, an infectious virus could not be detected in the blood samples of the ASFV-G-∆I177L/∆EP402R-inoculated animals in either group at any of the time points tested. Furthermore, while all of the mock-inoculated animals presented a quick and lethal clinical form of ASF after the intramuscular inoculation challenge with 10^2^ HAD_50_ of highly virulent parental field isolate Georgia 2010 (ASFV-G), all of the ASFV-G-∆I177L/∆EP402R-inoculated animals were protected, remaining clinically normal until the end of the observational period. Most of the ASFV-G-∆I177L/∆EP402R-inoculated pigs developed strong virus-specific antibody responses against viral antigens, reaching maximum levels at 28 days post inoculation. Importantly, all of the sera collected at that time point in the ASFV-G-∆I177L/∆EP402R-inoculated pigs did not react in a direct ELISA coated with the recombinant EP402R protein. Conversely, the EP402R protein was readily recognized by the pool of sera from the animals immunized with recombinant live attenuated vaccine candidates ASFV-G-∆I177L, ASFV-G-∆MGF, or ASFV-G-∆9GL/∆UK. Therefore, ASFV-G-∆I177L/∆EP402R is a novel, safe and efficacious candidate with potential to be used as an antigenically DIVA vaccine.

## 1. Introduction

The African swine fever virus (ASFV) is the causative agent of African swine fever (ASF), a lethal disease, which was first identified in 1921 in Kenya [1]. ASFV is a large, structurally complex virus, harboring a large, double-stranded DNA genome of 180–190 kilobase (kb) pairs, which encodes more than 160 genes [2]; recently, all of these genes have been predicted structurally [3] and the proteome of ASFV was determined [4,5]. We recently reported that there are six unique genotypes of ASFV [6], with the pandemic genotype 2 strain (previously classified as genotype II) gaining the most attention, as it is currently causing diseases in domestic and wild boar in Europe, Asia, Western Africa, and the island of Hispaniola [7,8,9,10,11,12]. The genotype 2 strains are highly virulent and are severely affecting pork production across the globe. As commercial vaccines for genotype 2 have become available in Vietnam only recently, disease control is still mostly implemented by culling all of the infected animals and strictly limiting the movement of susceptible pigs [13].

Several experimental recombinant live attenuated vaccine candidates have recently been developed via genetic manipulation, by deleting the ASFV genes involved in virulence in pigs. In general, these vaccine candidates were efficacious in protecting animals against the clinical disease caused by the virulent parental field isolate [14,15,16,17,18,19,20,21,22,23,24,25].

The ASFV-G-∆I177L strain, developed via the partial deletion of the I177L gene, is a safe and highly efficacious vaccine which has recently been approved for commercial use in Vietnam [14,15,26,27]. It is a desirable characteristic in commercial vaccines that the immune response that they elicit may be differentiated from that induced by the infection with virus field isolates (DIVA capability) in order to facilitate the epidemiological management needed to control or eradicate the disease in either disease-free or endemic areas. A proof-of-concept genetic DIVA test based on qPCR to differentiate animals vaccinated with ASFV-G-∆I177L from those infected with the parental virus ASFV Georgia 2010 isolate (ASFV-G) has been recently reported [28]. However, no serological DIVA test has been developed to differentiate animals that have received ASFV-G-∆I177L from those infected with ASFV field isolates. Here, we report the development of a novel ASFV-G-∆I177L, ASFV-G-∆I177L/∆EP402R, harboring the deletion of the EP402R gene that encodes for an antigenic envelope protein, the CD2 homolog, which mediates the adhesion to swine erythrocytes. Deletion of EP402R from ASFV-G resulted in no phenotypic change in virus virulence [21,29]. ASFV-G-∆I177L/∆EP402R replicates well in swine macrophages and remains free of residual virulence when IM inoculated to domestic pigs, even to doses as high as 10^6^ HAD_50_. Furthermore, ASFV-G-∆I177L/∆EP402R induced protection against the appearance of a clinical disease in animals that were inoculated with a dose as low as 10^2^ HAD_50_ and challenged with virulent parental ASFV-G. Importantly, a differential antibody response against the EP402R protein is absent in all ASFV-G-∆I177L/∆EP402R-inoculated pigs, which otherwise developed strong virus-specific antibody responses. Therefore, ASFV-G-∆I177L/∆EP402R constitutes a potential DIVA-compatible, novel, safe and efficacious vaccine candidate.

## 2. Materials and Methods

### 2.1. Viruses and Cells

Primary cell cultures of swine macrophages were obtained as previously described [30]. Macrophages were seeded at a concentration of 5 × 10^6^ cells/mL of culture media. Growth kinetic studies were comparatively performed between the original ASFV-G-∆I177L, ASFV-G-∆I177L/∆EP402R, and the parental virulent strain ASFV-G, using an MOI of 0.01 HAD_50_ or TCID_50_, as previously described [31], with sample points obtained at 2, 24, 48, 72, and 96 h post infection (p.i.). Virus titrations were performed using swine macrophage cultures in 96-well plates, as previously described [30]. Virus-infected cells were detected by either the presence of fluorescence (for ASFV-G-∆I177L/∆EP402R) or hemadsorption (HA) (for ASFV-G-∆I177L and ASFV-G). The virus titers were calculated using the Reed and Muench method [32,33].

The ASFV-G-∆I177L strain was previously developed in our laboratory [14]. ASFV-G-∆I177L/∆EP402R was developed by deleting the EP402R gene from the genome of ASFV-G-∆I177L by homologous recombination following routinary procedures [31]. The genetic structure of the recombinant plasmid is described in detail in a previous report [29], with the exception that instead of the βGUS gene, the green fluorescent gene (GFP) was used as the reporter gene.

### 2.2. Sequencing and Analysis of the ASFV-G-∆I177L/∆EP402R Genome

Viral DNA from the infected macrophage cultures that showed 90–100% CPE was obtained using the Nuclear Extract Kit (Active Motif, Carlsbad, CA, USA). After separation from the nucleus, the cytoplasmic fraction was used to obtain the viral DNA by following the manufacturer’s protocol. Briefly, virus-infected cells were harvested and treated with hypotonic buffer (20 mM Tris-HCl, pH 7.4; 10 mM NaCl; 3 mM MgCl_2_) on ice for 15 min (or until the cell membrane was dissolved). Then, the fraction containing the nucleus was separated by centrifugation, the cytoplasmic fraction was collected, and the DNA was extracted by adding 10% 3M NaOAc by volume to the sample (Sigma-Aldrich, St. Louis, MO, USA) and an equal volume of phenol–chloroform–isoamyl alcohol (25:24:1) with a pH of 6.5–6.9 (Sigma-Aldrich). These were then centrifuged at max speed in a tabletop centrifuge. Then, the aqueous phase was precipitated using 2 volumes of 100% ethanol, washed with the same volume of 70% ethanol, and dried. The obtained pellet of DNA was then resuspended in sterile water. The DNA library was then used for NGS sequencing using a Nextera XT kit in the NextSeq sequencer (Illumnia, San Diego, CA, USA), strictly following the manufacturer’s protocol. Sequence analysis was performed using CLC Genomics Workbench software (CLCBio, Waltham, MA, USA).

### 2.3. Detection of ASFV and EP402R Protein-Specific Antibodies

ASFV antibody detection was performed using an in-house ELISA previously described [34]. Briefly, the ELISA antigen was prepared from ASFV-infected Vero cells. Maxisorb ELISA plates (Nunc, St Louis, MO, USA) were coated with 1 µg per well of infected or uninfected cell extract. The plates were blocked with phosphate-buffered saline containing 10% skim milk (Merck, Kenilworth, NJ, USA) and 5% normal goat serum (Sigma, Saint Louis, MO, USA). Each swine serum was tested at multiple dilutions against both the infected and uninfected cell antigen. ASFV-specific antibodies in the swine sera were detected using an anti-swine IgG-horseradish peroxidase conjugate (KPL, Gaithersburg, MD, USA) and SureBlue Reserve peroxidase substrate (KPL, Milford, MA, USA). Plates were read at OD630 nm in an ELx808 plate reader (BioTek, Shoreline, WA, USA). Sera titers were expressed as the log10 of the highest dilution where the OD630 reading of the tested sera at least duplicated the reading of the mock-infected sera.

The EP402R protein was expressed using a commercially available baculovirus system following the previously described routinary procedures [35]. Briefly, a truncated EP402R open-reading frame was amplified using the purified viral DNA from the ASFV Georgia 2007/1 isolate with primer set 5′-AGTCCACCACCTGAATCTAATGAA G-3′, and 5′-TGGCGGGATATTGGGTAGTAGC-3′. The amplicon was cloned into baculovirus using the Bac-to-Bac HBM TOPO Secreted Expression system (ThermoFisher Scientific, Waltham, MA, USA). The expression and purification of the recombinant antigen was performed as previously described [35]. Maxisorb ELISA plates (Nunc, St Louis, MO, USA) were coated with 0.1 µg per well of baculovirus-expressed EP402R protein. The plates were blocked with phosphate-buffered saline containing 10% skim milk (Merck, Kenilworth, NJ, USA). Swine sera were tested, their reactivity detected, and the titers calculated as described above for the anti-ASFV antibodies.

### 2.4. Evaluation of Virulence and Efficacy of ASFV-G-∆I177L/∆EP402R in Domestic Pigs

The virulence of the recombinant ASFV-G-∆I177L/∆EP402R was evaluated in 35–40 kg commercial-breed pigs. Groups of five pigs were experimentally infected via the intramuscular route (IM infected) with either 10^2^ or 10^6^ TCID_50_ of ASFV-G-∆I177L/∆EP402R. The appearance of clinical signs (such as depression, anorexia, staggering gait, purple skin discoloration, diarrhea, and coughing), as well as changes in body temperature, were recorded daily throughout the experiment. Blood and serum samples were taken at days 0, 4, 7, 11, 14, 21, and 28 p.i.

Efficacy studies were performed by intramuscularly (IM) inoculating the animals 28 days post ASFV-G-∆I177L/∆EP402R inoculation with 10^2^ HAD_50_ of virulent parental ASFV-G. An additional group of 5 naïve animals were incorporated as a control. All animals were observed daily for 21 days, sampled, and recorded as described above.

All animal experiments were performed under biosafety level 3 conditions in the animal facilities at Plum Island Animal Disease Center, strictly following a protocol approved by the Institutional Animal Care and Use Committee (225.06-19-R_090716, approved on 9 June 2019).

## 3. Results

### 3.1. Development of ASFV-G-∆I177L/∆EP402R

**Figure 1:**
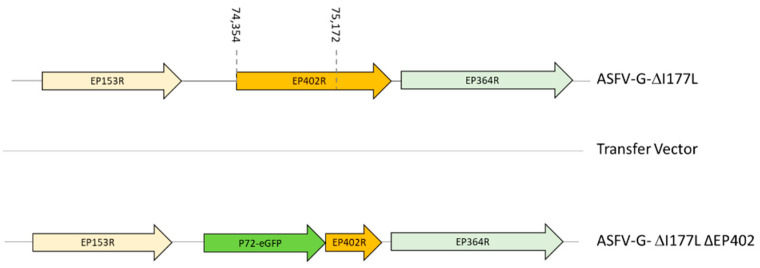
Schematic for the development of ASFV-G-∆I177L/∆EP402R. The transfer vector contains the p72 promoter and the GFP gene (the gene positions are indicated). The homologous arms were designed to have flanking ends to both sides of the deletion/insertion cassette. The nucleotide positions of the area that was deleted in the ASFV-G genome are indicated with the dashed lines.

ASFV-G-∆I177L was used as parental virus that was constructed as previously described [14], and was used to develop ASFV-G-∆I177L/∆EP402R by substituting nucleotides 10–1083 of the EP402R gene with p72GFP and using methodologies that rely on homologous recombination, which have been previously described [31]. ASFV-G-∆I177L/∆EP402R contains a deletion of 819-bp (nucleotide positions 74,354–75,172 from the ASFV-G-∆I177L/∆EP402R virus). This area was replaced with a cassette containing the p72GFP cassette (Figure 1).

The recombinant purified virus was obtained after 12 passages of successive limiting dilution events in the primary swine macrophage cell cultures. The accuracy of the genetic modifications introduced in ASFV-G-∆I177L to develop ASFV-G-∆I177L/∆EP402R were evaluated by sequencing the whole virus genome via next-generation sequencing (NGS). The genome analysis confirmed the accuracy of the introduced modifications, the absence of any additional mutations, and the absence of the parental genome in the ASFV-G-∆I177L/∆EP402R stock.

### 3.2. Evaluation of Replication of ASFV-G-∆I177L/∆EP402R

The growth kinetic of ASFV-G-∆I177L/∆EP402R was evaluated in comparative studies with its parental virus, ASFV-G-∆I177L, and the virulent field isolate ASFV-G. This study was conducted on primary macrophage cell cultures, performing a multistep growth curve using infections at a low MOI (0.01). Virus yields were evaluated at 2, 24, 48, 72, and 96 h post infection by titrating the samples in the primary swine macrophages.

**Figure 2:**
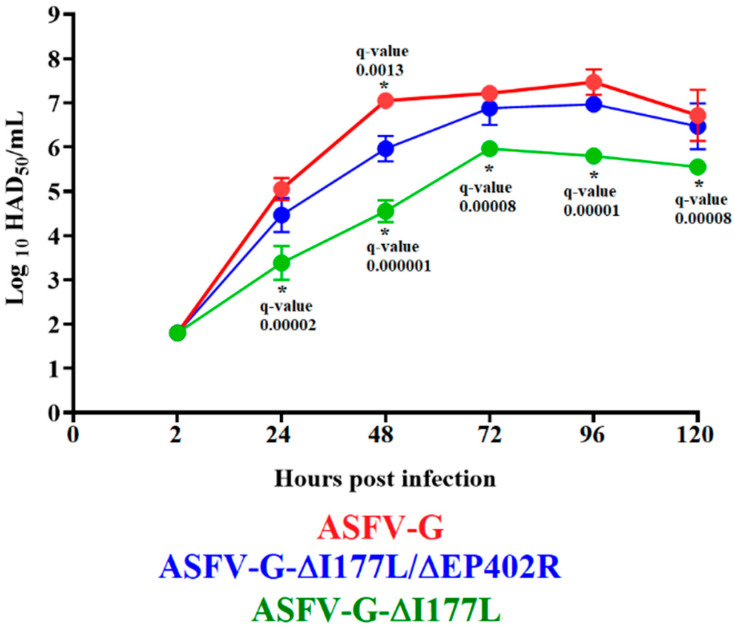
In vitro growth kinetics of ASFV-G-∆I177L/∆EP402R, ASFV-G-∆I177L, and ASFV-G in primary swine macrophage cell cultures (MOI = 0.01). Data represent means and standard deviations. The sensitivity using this methodology for detecting viruses is ≥10^1.8^ HAD_50_ or TCID_50_/mL. An unpaired t-test using the two-stage step-up (Benjamini, Krieger, and Yekutieli) method was used to assess statistical differences in viral yields between ASFV-G-∆I177L/∆EP402R and either ASFV-G or ASFV-G-∆I177L at different time points. The significance of these discoveries was evaluated using the false discovery rate method (FDR), with q-values < 0.05 (*) considered significant.

Results showed that ASFV-G-∆I177L/∆EP402R exhibits almost indistinguishable kinetics of replication when compared to ASFV-G, since no statistical differences could be established in the virus yields between these two viruses in any of the time points evaluated save for the data point at 48 h p.i. (Figure 2). However, ASFV-G-∆I177L showed a slight, but statistically significant, decrease in replicative ability when compared with ASFV-G-∆I177L/∆EP402R. Differences between ASFV-G-∆I177L and ASFV-G-∆I177L/∆EP402R varied from 10^0.5^ to 10^1^ TCID_50_ or HAD_50_/mL, depending on the time point p.i. considered. Therefore, deletion of the EP402R gene from the genome of ASFV-G-∆I177L seems to slightly improve its ability to replicate in swine macrophages.

### 3.3. Assessment of ASFV-G-∆I177L/∆EP402R Replication and Potential Residual Virulence in Domestic Pigs

**Figure 3:**
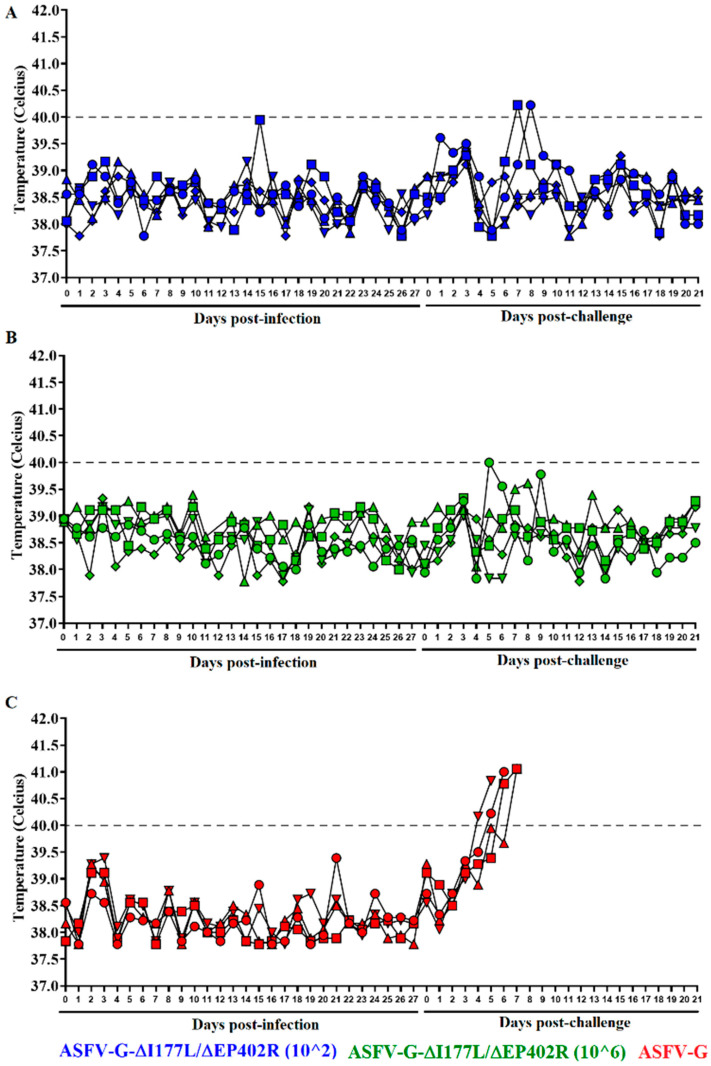
Evolution of body temperature in animals IM inoculated with either (**A**) 10^2^ or (**B**) 10^6^ TCID_50_ of ASFV-G-∆I177L/∆EP402R, or (**C**) mock-inoculated, and challenged 28 days later with 10^2^ HAD_50_ of parental virulent ASFV-G. Data points represent individual animals.

To evaluate the efficacy of the replication of ASFV-G-∆I177L/∆EP402R and the potential presence of residual virulence in domestic pigs, different groups of five 35–40 kg pigs were IM inoculated with either 10^2^ or 10^6^ TCID_50_ of ASFV-G-∆I177L/∆EP402R or mock-inoculated. The presence of clinical signs associated with ASF were then monitored daily for 28 days post inoculation. Animals inoculated with either dose of ASFV-G-∆I177L/∆EP402R showed the same clinical behavior as the mock-inoculated animals. The recordings of the rectal temperature values in all animals of the three groups remained within a normal range (not higher than 40 °C) during the observational period (Figure 3). Therefore, similar to its parental ASFV-G-∆I177L [14], ASFV-G-∆I177L/∆EP402R remains completely attenuated to domestic pigs even when inoculated with viral doses as high as 10^6^ TCID_50_.

**Figure 4:**
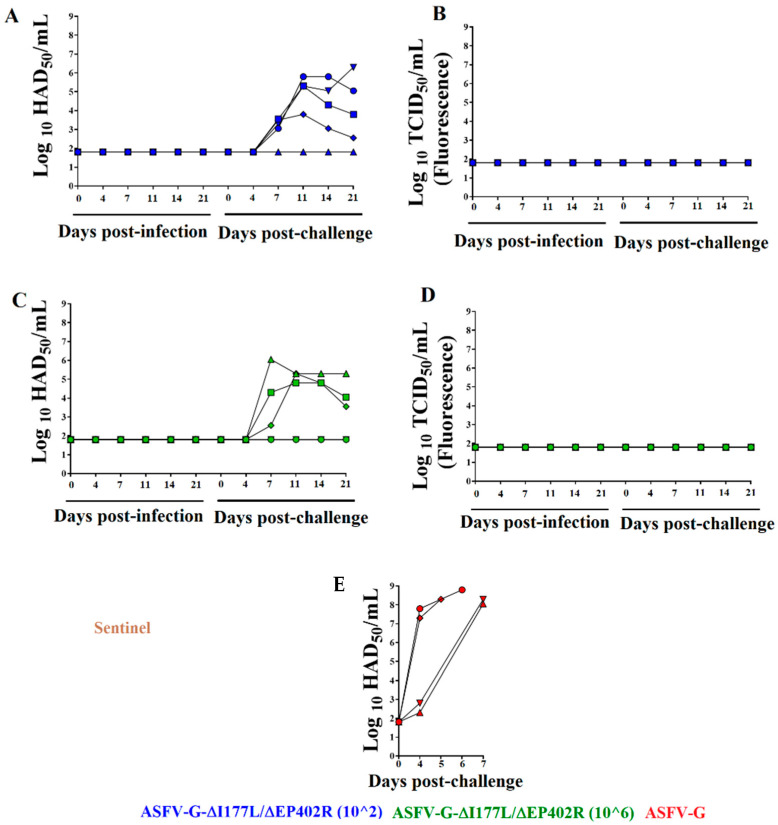
Viremia titers detected in animals IM inoculated with either (**A**,**B**) 10^2^ or (**C**,**D**) 10^6^ TCID_50_ of ASFV-G-∆I177L/∆EP402R, or (**E**) mock-inoculated and challenged 28 days later with 10^2^ HAD_50_ of parental virulent ASFV-G. Titer values for ASFV-G-∆I177L/∆EP402R and for the ASFV-G strains were calculated based on the detection of infected cells by the presence of fluorescence or HA, respectively. Data points represent individual animals. Sensitivity of virus detection: ≥10^1.8^ HAD or TCID_50_/mL.

The replication of recombinant ASFV-G-∆I177L/∆EP402R in the inoculated animals was analyzed by assessing viremia titers at different times post inoculation. The detection of infected macrophages in the 96-well plates used in the titration procedure was based on the presence of fluorescence. Interestingly, viremia titers in the animals inoculated with either 10^2^ or 10^6^ TCID_50_ remained undetectable for all animals during the 28-day post-inoculation period (Figure 4). The sensitivity of the detection of infectious virus in these clinical samples is ≥10^1.8^ TCID_50_/mL; so, in order to confirm the absence of virus, DNA samples were subjected to a qPCR test specifically designed to detect the presence of the ASFV p72 gene. To increase detection sensitivity, all clinical blood samples were subjected to a passage in primary swine macrophage cultures for 5 days and the resulting material was evaluated using qPCR, as described elsewhere [28]. Therefore, removal of the EP402R gene from the ASFV-G-∆I177L drastically affects the ability of the resulting virus, ASFV-G-∆I177L/∆EP402R, to systemically replicate after the parenteral inoculation in domestic pigs. Conversely, ASFV-G-∆I177L has repeatedly been shown to reach significant viremia titers between days 4 and 11 post intramuscular inoculation [14,15,27,36]. It is interesting to note that although ASFV-G-∆I177L/∆EP402R showed a slight increased replication over the parental ASFV-G-∆I177L in the primary swine macrophage cultures, it appears to have a drastically decreased ability to replicate when experimentally inoculated in pigs.

### 3.4. Efficacy of ASFV-G-∆I177L/∆EP402R to Protect Domestic Pigs against the Challenge of the Virulent Parental ASFV-G

Defining the immune host mechanism involved in the process of protection against virulent field strains of ASFV is still a controversial issue [37,38,39]. Although some correlation was attempted to be established between the presence of T cell responses and protection, our results showed that the main factor regularly associated with protection against ASFV infection is the level of virus-specific circulating antibodies [40]. The evaluation of the levels of ASFV-specific serum antibodies in both groups of animals inoculated with ASFV-G-∆I177L/∆EP402R were performed using an in-house-developed ELISA [34].

**Figure 5:**
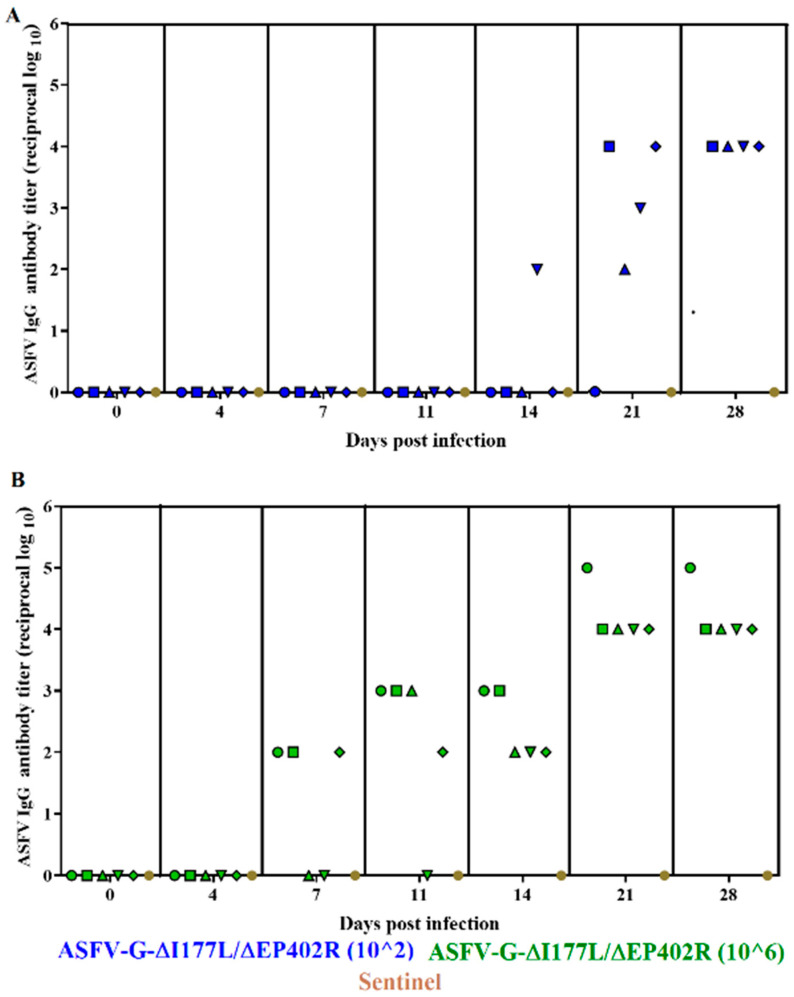
Anti-ASFV antibody titers detected via an ELISA in pigs IM inoculated with either (**A**) 10^2^ or (**B**) 10^6^ TCID_50_ of ASFV-G-∆I177L/∆EP402R. Each point represents values from individual animals. Titers are expressed as the log_10_ of the inverse of the highest serum dilution that still duplicates OD of the pre-inoculation serum.

All animals inoculated with 10^2^ TCID_50_ of ASFV-G-∆I177L/∆EP402R (with the exception of one that remained unresponsive) started developing a clear virus-specific antibody response by day 21 p.i., reaching the highest titers by day 28 p.i. (Figure 5). The group of animals inoculated with 10^6^ TCID_50_ of ASFV-G-∆I177L/∆EP402R presented an earlier presence of circulating antibodies. Three of the animals presented detectable levels by day 7 p.i., four of them were positive by day 11, and all were positive by day 14 p.i. Titers reached maximum levels by day 21 p.i., remaining at that level until the last day of the challenge, at 28 days p.i. Therefore, most animals infected with ASFV-G-∆I177L/∆EP402R, regardless of the dose of virus they received, developed a strong antibody response by the end of the challenge.

Furthermore, to evaluate the efficacy of ASFV-G-∆I177L/∆EP402R in mediating protection after the challenge with the highly virulent parental ASFV-G strain, the two groups of pigs inoculated 28 days earlier with either 10^2^ or 10^6^ TCID_50_/mL of ASFV-G-∆I177L/∆EP402R were IM inoculated with 10^2^ HAD_50_ of ASFV-G. The mock-vaccinated animals were also inoculated, under identical conditions, as a control group.

**Figure 6:**
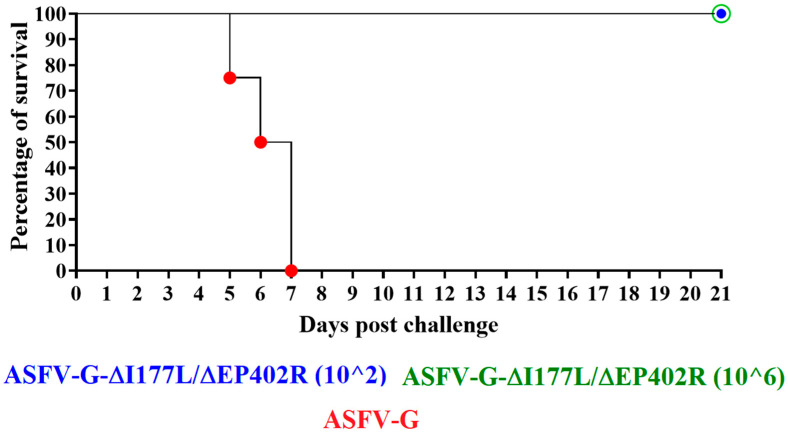
Evolution of mortality in animals (five animals/group) IM inoculated with either 10^2^ or 10^6^ TCID_50_ of ASFV-G-∆I177L/∆EP402R, or mock-vaccinated (four animals/group) and challenged 28 days later with 10^2^ HAD_50_ of parental virulent ASFV-G.

Animals in the mock-vaccinated group started presenting ASFV clinical signs by days 4–6 post challenge, quickly evolving to a severe form of the disease, with these pigs needing to be euthanized between days 5 and 7 post challenge (p.c.) (Figure 3 and Figure 6). In opposition, the animals inoculated with ASFV-G-∆I177L/∆EP402R did not present clinical signs associated with ASF and continued to be clinically normal during the observation period of 21 days (Figure 3 and Figure 6). Two animals in the group inoculated with 10^2^ TCID_50_/mL, at days 7 and 8 p.c., and one animal inoculated with 10^6^ TCID_50_/mL, at day 5 p.c., presented one-day rises in their body temperature, reaching or slightly exceeding 40 °C (Figure 3). These transitory rises in body temperature were not associated with any other clinical sign associated with ASF. Therefore, inoculation with ASFV-G-∆I177L/∆EP402R produced protection against the challenge of the virulent parental virus, solidly preventing the appearance of clinical ASF disease.

Replication of the challenge virus was monitored by determining viremia titers: specifically by detecting the presence of ASFV-G-infected cells using HA (since ASFV-G-∆I177L/∆EP402R is not able to mediate HA). ASFV-G titer values in the blood of the challenged control animals were detected by the 4th day p.c., with relatively low values in two of the animals (in the range of 10^2–3^ HAD_50_/mL) and higher values in the other two (in the range of 10^7–8^ HAD_50_/mL), increasing to high titers in all of them (in the range of 10^7.5–8.5^ HAD_50_/mL) by the time they were euthanized on day 6–7 p.c. due to the severity of their clinical symptoms.

All but one of the five animals inoculated with 10^2^ TCID_50_ of ASFV-G-∆I177L/∆EP402R developed ASFV-G viremias by day 4 p.c., in the range of 10^3–4^ HAD_50_/mL, which persisted with a variety of titers (in the range of 10^2.5–6.5^ HAD_50_/mL) until the end of the observational period at 21 days p.c. In addition, no ASFV-G-∆I177L/∆EP402R viremia titers were detected in these animals from the time of the challenge until the end of the experiment. Similarly, three animals receiving 10^6^ TCID_50_ of ASFV-G-∆I177L/∆EP402R developed ASFV-G viremias detected by day 7 p.c., with titers in the range of 10^3.5–5.5^ HAD_50_/mL, which all lasted until the end of the experiment. The other two animals did not show detectable ASFV-G titers in their blood. Also, no ASFV-G-∆I177L/∆EP402R viremia titers were detected from the start of the challenge until the end of the challenge in these animals inoculated with 10^6^ TCID_50_ of ASFV-G-∆I177L/∆EP402R.

### 3.5. Evaluation of ASFV-G-∆I177L/∆EP402R as a Potential Antigenic Marker Vaccine

**Figure 7:**
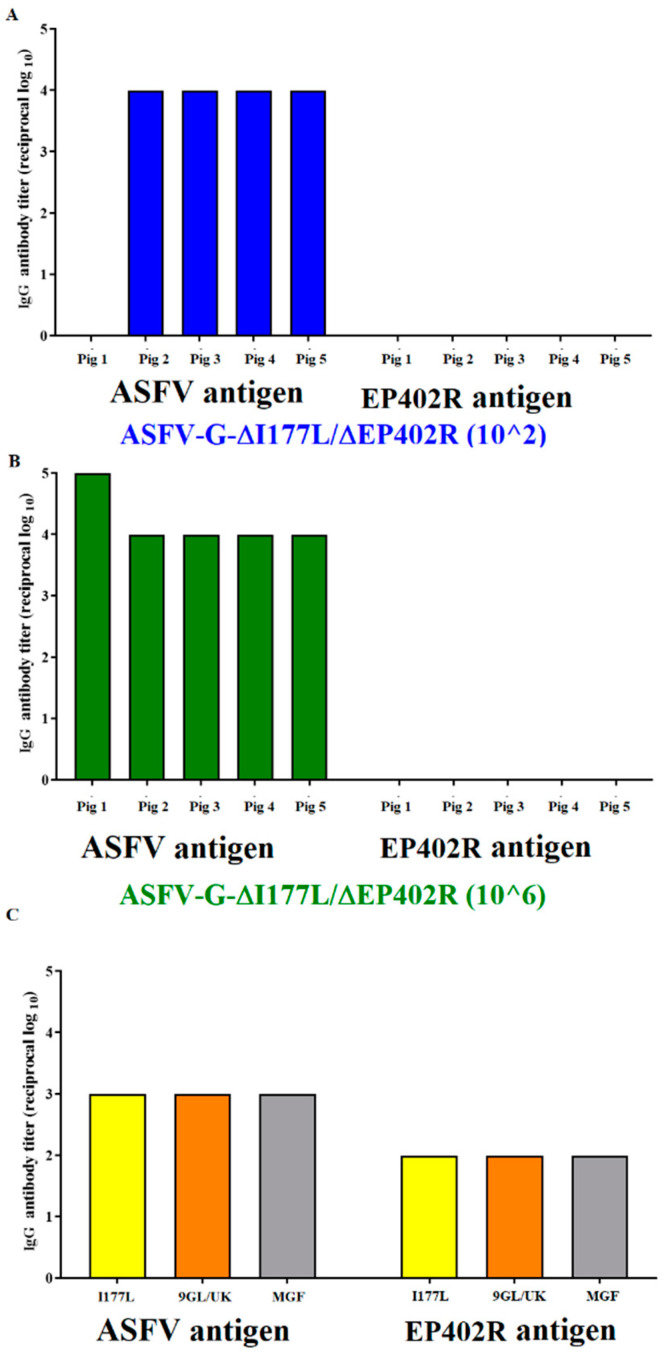
Anti-ASFV and EP402R protein antibody titers detected via an ELISA in the sera of pigs IM inoculated with either (**A**) 10^2^ or (**B**) 10^6^ TCID_50_ of ASFV-G-∆I177L/∆EP402R. Each data point represents values from individual animals. (**C**) Anti-ASFV and EP402R protein antibody titers detected via ELISA in pools of sera from animals immunized with recombinant vaccine candidates ASFV-G-∆I177L, ASFV-G-∆9GL/∆UK, or ASFV-G-∆MGF. Titers are expressed as the log_10_ of the inverse of the highest serum dilution that still duplicates OD of the pre-inoculation serum.

As shown above, most of the animals inoculated with ASFV-G-∆I177L/∆EP402R had a strong ASFV-specific antibody response by the end of challenge (Figure 5). It was important, then, to evaluate if their reactivity to the EP402R protein could constitute the basis for the development of a DIVA test that would differentiate the systemic antibody response of animals inoculated with ASFV-G-∆I177L/∆EP402R from those infected with viruses harboring the EP402R gene. An in-house ELISA was used to detect the presence of anti-EP402R protein-specific antibodies (see the Materials and Methods section). The results demonstrated that all sera from the animals inoculated with ASFV-G-∆I177L/∆EP402R, obtained 28 days post infection, failed to recognize the EP402R protein (Figure 7). Conversely, sera obtained 28 days post infection from the animals with the recombinant ASFV vaccine candidates (ASFV-G-∆I177L, ASFV-G-∆9GL/∆UK, or ASFV-G-∆MG) reacted strongly against both the ASFV antigens and the EP402R protein. These results demonstrate that ASFV-G-∆I177L/∆EP402R functions as an antigenically marked vaccine, specifically not inducing antibodies to the EP402R protein, which is otherwise easily recognized by animals infected with viruses harboring the EP402R gene.

## 4. Discussion

In this report, we evaluated the added deletion of the EP402R gene in the vaccine candidate ASFV-G-∆I177L as a potential negative antigenic marker to differentiate the antibody response between vaccinated and infected animals (DIVA concept). Longer-term studies will have to be performed to determine the feasibility of detecting antibodies to EP402R in the longer term after vaccination with vaccines containing DP402R or with animals surviving natural infection with ASFV. A similar construct has been previously reported for the Chinese GZ201801 isolate, wherein a similar virus, ASFV-GZ∆I177L∆CD2v, was constructed with a deletion of both the I177L and EP402R genes [41]. Although the ASFV-G-∆I177L/∆EP402R and SFV-GZ∆I177L∆CD2v constructs are nominally similar, several phenotypic characteristics are different between these two viruses. It should be noted that the designs of the I177L gene deletion in these two viruses are different. In GZ201801, the deletion resulted in the N-terminal 111 amino acid portion of I177L being produced [41], while in our ASFV-G-∆I177L, the deletion was designed to produce only a theoretical 25-amino-acid product, matching the first 18 residues in the I177L ORF before a frameshift occurs, plus an additional 7 amino acid residues being produced before a stop codon [14]. Therefore, unlike the deletion that was produced in the GZ201801 isolate, in our ASFV-G-∆I177L, the I177L gene would be functionally blocked, as only the transmembrane region with nonsense amino acids would be produced, whereas in the GZ201801, an additional 76 amino acids are produced following the transmembrane region—likely causing the ASFV-GZ∆I177L to retain at least part of I177L’s function. This is likely the cause of the ASFV-GZ∆I177L’s retention of residual virulence when administered IM at a dose of 10^5^ TCID_50_, inducing fever and joint inflammation in the inoculated animals, unlike previous reports stating that ASFV-G-∆I177L completely lacks residual virulence when administered at a high dose (10^6^ HAD_50_) [14,26], even in young animals [36]. In addition, the deletion of the EP402R gene was also performed differently in our study, wherein the first 819 nucleotides (encoding for the first 273 amino acids of the protein) were deleted from the EP402R gene to ensure the preservation of the promoter region of the neighboring gene EP364R. As designed here, the residual EP402R gene has no promoter after the insertion cassette, and is not produced, thus leaving Ep402R functionally deleted. However, in the GZ201801 isolate, the entire ORF of 1082 nucleotides for EP402R was removed; this results in a very short promoter sequence for the Ep364R gene, which could potentially affect the expression of the Ep364R gene in the GZ201801 isolate. The difference in the design of the deletions of both the I177L and the EP402R genes could also explain why the results on the protective efficacy of ASFV-G-∆I177L/∆EP402R differ to those reported for AFV-GZ∆I177L∆CD2v, where most of the animals immunized with that virus developed joint swelling after being challenged with the parental virus [41].

The addition of EP402R deletion to the ASFV-G-∆I177L vaccine candidate appears to result in a decrease in the protective efficacy when compared with that of the ASFV-G-∆I177L vaccine alone, wherein, at 10^2^ TCID_50_, vaccinated animals at days 7 and 8 p.c., and one animal inoculated with 10^6^ TCID_50_ at day 5 p.c., presented one-day rises in their body temperature reaching or slightly exceeding 40 °C (Figure 3), which was not observed in the initial studies using ASFV-G-∆I177L. However, it should be stressed that no other clinical sign associated with ASF occurred, suggesting that ASFV-G-∆I177L/∆EP402R is a viable vaccine candidate producing protection against the challenge of the virulent parental virus and solidly preventing the appearance of clinical ASF disease. However, in some of the animals receiving 10^6^ TCID_50_ of ASFV-G-∆I177L/∆EP402R, after the challenge, replication of ASFV-G occurred, an observation that was not observed during the ASFV-G-∆I177L studies. Further studies will be needed to clarify if the presence of a challenge virus in those animals could have any negative effects, such as virus shedding and transmission to susceptible hosts. With the recent approval for commercial use of ASFV-G-∆I177L in Vietnam, after rigorous safety and efficacy field trials, ASFV-G-∆I177L/∆EP402R will require similar studies before it can be considered for approval for use in commercial farms.

As research groups worldwide further develop experimental vaccines for ASFV, it is important that each individual vaccine be considered a new vaccine and that rigorous testing is performed. A recent report from the World Organization for Animal Health (WOAH) specified that illegal production and use of ASF vaccines can cause serious epidemiological problems. As we highlighted in this report, differential deletion designs of the I177L gene could be the cause of the presence of residual virulence that was observed in ASFV-GZ-∆I177L, which was not observed when the I177L gene was deleted correctly as we initially designed in ASFV-G-∆I177L.

It is important that research groups conduct adequate safety and efficacy testing to fully understand the characteristics of new live attenuated vaccine candidates. As we have shown here, although ASFV-G-∆I177L/∆EP402R is a safe vaccine, there are some differences between this vaccine candidate and the original ASFV-G-∆I177L. We have previously attempted to add a DIVA marker to ASFV-G-∆I177L through deletion of the ASFV gene E184L, which was a known, highly immunogenic protein that, when individually deleted, was shown to partially attenuate ASFV-G; however, when added to the ASFV-G-∆I177L vaccine platform, it resulted in the loss of vaccine efficacy [42]. A similar observation was made when another potential immunogenic protein, MGF110-5L-6L, was deleted from the ASFV-G-∆I177L vaccine platform. In this case, MGF110-5L-6L, when deleted on its own from ASFV-G, did not attenuate ASFV-G; however, when deleted from the ASFV-G-∆I177L backbone, there was also a loss of vaccine efficacy [36]. Here, we reported that successful deletion of the DIVA marker EP402R from the ASFV-G-∆I177L vaccine retained the vaccine efficacy of the parental ASFV-G-∆I177L. Interestingly, when EP402R was deleted from a different vaccine candidate, ASFV-G-∆9GL [43], producing recombinant ASFV-G-∆9GL∆CD2 [44], the vaccine efficacy was lost. As we have observed here, having the correct deletion combinations in live attenuated vaccines is important to maintain vaccine safety and efficacy in the development of future live attenuated vaccines for ASF.

## Data Availability

All data are included in the manuscript.
